# De Novo Antimicrobial Peptide Design with Feedback Generative Adversarial Networks

**DOI:** 10.3390/ijms25105506

**Published:** 2024-05-18

**Authors:** Michaela Areti Zervou, Effrosyni Doutsi, Yannis Pantazis, Panagiotis Tsakalides

**Affiliations:** 1 Department of Computer Science, University of Crete, 700 13 Heraklion, Greece; 2 Institute of Computer Science, Foundation for Research and Technology-Hellas, 700 13 Heraklion, Greece; edoutsi@ics.forth.gr; 3 Institute of Applied and Computational Mathematics, Foundation for Research and Technology-Hellas, 700 13 Heraklion, Greece; pantazis@iacm.forth.gr

**Keywords:** antimicrobial peptides (AMP), protein function classification, gated recurrent neural networks, *k*-mers, protein transfer learning, generative adversarial networks

## Abstract

Antimicrobial peptides (AMPs) are promising candidates for new antibiotics due to their broad-spectrum activity against pathogens and reduced susceptibility to resistance development. Deep-learning techniques, such as deep generative models, offer a promising avenue to expedite the discovery and optimization of AMPs. A remarkable example is the Feedback Generative Adversarial Network (FBGAN), a deep generative model that incorporates a classifier during its training phase. Our study aims to explore the impact of enhanced classifiers on the generative capabilities of FBGAN. To this end, we introduce two alternative classifiers for the FBGAN framework, both surpassing the accuracy of the original classifier. The first classifier utilizes the *k*-mers technique, while the second applies transfer learning from the large protein language model Evolutionary Scale Modeling 2 (ESM2). Integrating these classifiers into FBGAN not only yields notable performance enhancements compared to the original FBGAN but also enables the proposed generative models to achieve comparable or even superior performance to established methods such as AMPGAN and HydrAMP. This achievement underscores the effectiveness of leveraging advanced classifiers within the FBGAN framework, enhancing its computational robustness for AMP de novo design and making it comparable to existing literature.

## 1. Introduction

Antimicrobial peptides (AMPs) are small proteins that are naturally produced by many organisms, including humans, as part of their innate immune response to infections [1]. AMPs are promising candidates for new antibiotics, as they have broad-spectrum activity against a wide range of pathogens and are less prone to resistance development [2]. However, the design and optimization of synthetic AMPs present formidable challenges. The vast sequence space of potential peptide candidates, coupled with the complex interplay between sequence, structure, and function, make traditional experimental approaches to AMP discovery time-consuming and resource-demanding [3]. In this context, the introduction of deep-learning techniques provides a promising opportunity to speed up the discovery of AMPs [4,5].

**Figure 1:**
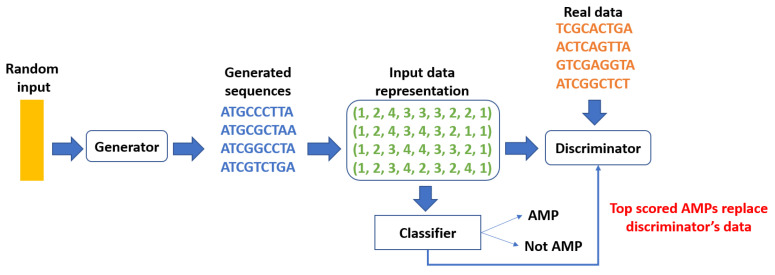
General FBGAN pipeline. The feedback loop during training replaces real data with synthetic data over time.

In recent years, there has been significant interest in utilizing deep-learning techniques for developing new antimicrobial peptides (AMPs) [6,7,8,9]. A notable study by Gupta and Zou [6] demonstrated successful AMP design through Generative Adversarial Networks (GANs) [10], specifically introducing the Feedback GAN (FBGAN) model. As depicted in Figure 1, FBGAN generates synthetic DNA sequences encoding proteins with antimicrobial properties, utilizing three neural networks: a generator, a discriminator, and a classifier. The generator creates synthetic peptide sequences while the discriminator evaluates them, distinguishing between real and generated peptides. Through adversarial training, the generator improves its ability to produce sequences indistinguishable from real peptides. Nevertheless, the critical aspect of the FBGAN framework for optimizing AMP functionality lies in the incorporation of a classifier. This classifier plays a pivotal role in guiding the training process toward generating sequences with a high likelihood of being AMPs. Specifically, it evaluates the generated sequences to determine whether they exhibit AMP properties or not. Sequences with scores above a threshold of 0.8 are fed back into the discriminator’s dataset, replacing previous sequences. This feedback mechanism plays a crucial role in directing the training process toward producing sequences with a high probability of being AMPs. However, determining the exact extent to which the classifier influences the quality and effectiveness of de novo peptide design remains a crucial question. Hence, this study aims to *investigate the pivotal role of the classifier by examining how its performance impacts the overall quality of newly designed peptides*.

In light of this objective, starting from the FBGAN classifier as our baseline, we propose two alternative classifiers, each surpassing the other in performance. This gradual approach enables us to assess how incremental improvements in classifier quality influence the de novo design. It is important to note that state-of-the-art AMP classifiers heavily rely on sophisticated feature extraction and selection techniques (e.g., [11,12,13,14,15,16,17,18,19]), along with complex architectures such as graph transformers (e.g., sAMPpred-GAT [20]). While these classifiers excel in accuracy, they are not well-suited for integration into generative models due to their heavy computational demands and complexity. In contrast, our objective is to utilize lightweight yet accurate classifiers that seamlessly integrate into the generative framework, such as the proposed classifier of FBGAN, which solely relies on primary sequence information. Notably, the FBGAN classifier employs Gated Recurrent Units (GRUs) [21] and a simplistic representation, encoding protein sequences into numerical sequences based on DNA base indices ({A:1, T:2, C:3, G:4}), potentially overlooking crucial structural and functional aspects (see Figure 1).

In this work, we explore two alternative classifiers that also rely solely on the primary sequence to provide more comprehensive representations capable of capturing the intricate structural and functional information within protein sequences, (i) we utilize the *k-mers technique* [22], a robust bioinformatics method that segments the sequence into subsequences of length *k*, effectively capturing local structural and functional properties, and (ii) we investigate *transfer learning* with Evolutionary Scale Modeling 2 (ESM2) [23], a pre-trained large protein language model. ESM2 is trained on extensive protein data, enabling it to learn representations or embeddings of protein sequences that capture rich structural and functional information. While previous studies have employed the k-mers technique on amino acid sequences [15,17], to the best of our knowledge, this work marks the first application of the *k*-mers technique specifically to DNA sequences for AMP classification. The evaluation results demonstrate that the *k*-mers representation surpasses the simplistic numerical encoding used in the FBGAN classifier, striking a better balance between model and data size. On the other hand, the ESM2 approach outperforms both the initial simplistic representation and the *k*-mers-based approach.

Subsequently, these classifiers are integrated into the FBGAN architecture, yielding two alternative models: FBGAN-kmers and FBGAN-ESM2. We evaluate the performance of these proposed models, comparing them not only against the original FBGAN but also against top-performing methods for AMP generation, namely AMPGAN [7] and HydrAMP [9]. Moreover, to ensure a fair and unbiased evaluation process in the performance of the generative models, we utilize external classifiers. In particular, the performance of each generative model in generating AMPs with high probability is validated through $CAMP_{R4}$ server [19]. This external platform for AMP classification is equipped with various classifiers, including Support Vector Machine (SVM), Random Forest (RF), and Artificial Neural Network (ANN) models. By leveraging these external classifiers, we aim to minimize biases that may arise from the excessive tuning of our models to specific training data, therefore enhancing their generalization to new or unseen data. This multi-classifier approach contributes to robustness and reliability in computationally predicting antimicrobial peptides. Overall, our assessment with $CAMP_{R4}$ demonstrates that both FBGAN-kmers and FBGAN-ESM2 exhibit superior performance compared to FBGAN, underscoring the significance of a more accurate classifier. Furthermore, the proposed alternative models outperform HydrAMP and compete favorably against AMPGAN.

The contributions of this paper are summarized below:

- Introduce two alternative classifiers based on *k*-mers and transfer learning with ESM2 for identifying AMPs, surpassing the baseline FBGAN classifier in performance.
- Propose two alternative generative models, FBGAN-kmers and FBGAN-ESM2, that confirm superior performance compared to FBGAN, highlighting the significance of incorporating advanced classifiers.
- Compare FBGAN-kmers and FBGAN-ESM2 with existing state-of-the-art methods for AMP generation, demonstrating competitive performance.

The rest of the paper is organized as follows: Section 2 presents the results of our comparative evaluation, including the performance of the proposed FBGAN-kmers and FBGAN-ESM2 classifiers compared to the baseline FBGAN classifier and the state-of-the-art generative models AMPGAN and HydrAMP. Section 3 discusses the implications of the results, highlighting the significance of classifier accuracy in de novo peptide design efficacy. Section 4 provides details on the materials, datasets, models, and methodologies used in the study, while Section 5 concludes with a summary of the key findings and suggestions for future research.

## 2. Results

### 2.1. Performance of Classifiers

**Figure 2:**
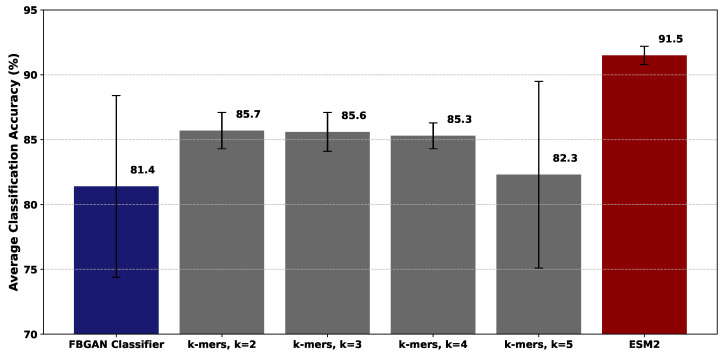
Average classification accuracy for each proposed model and FBGAN classifier. The standard deviation is reported in parentheses.

This section evaluates the performance of the proposed models in terms of classification accuracy. Each proposed model is measured against the FBGAN classifier. The results presented in Figure 2 indicate that for every *k*-value, the models outperform the FBGAN classifier. However, the performance of the model for k=5 is suboptimal. One potential reason the model exhibits poorer performance compared to the k=2, k=3, and k=4 models could be due to overfitting. As reported in Table 1, with over 200K parameters, the k=5 model may have too much capacity relative to the dataset’s size and could quickly memorize the training data instead of learning generalizable features. In addition to the poorer classification accuracy of the k=5 model, we also observed a higher variability in its performance across multiple runs. This suggests that this model is unable to generalize well to new data. It is worth noting that the complexity of the model should ideally align with the size of the dataset, ensuring a proportional relationship between model complexity and dataset scale. Thus, the observed limitations might stem from the dataset’s relatively modest size rather than inherent flaws in the model’s design.

On the contrary, the k=2, k=3, and k=4 models exhibit higher classification accuracy and lower variability, indicating that they are more stable and reliable in their predictions. Evidently, the *k*-mers-based models have significantly fewer parameters (see Table 1 and Section 4 for more details) than FBGAN classifier demonstrating not only the superior accuracy of the *k*-mers-based models but also the significance of minimizing unnecessary model complexity.

In terms of precision, recall, and F1-score, as presented in Table 2, the FBGAN classifier achieves a moderate performance with a relatively stable precision but exhibits a significant variability in the recall. This indicates that the classifier’s sensitivity to true positive instances is poor, which can potentially influence the overall success rate and efficiency of the de novo design of feedback-based generative models. On the contrary, the *k*-mers-based models consistently outperform the FBGAN classifier across all *k*-values for every metric, underscoring the superior performance of our approach. Notably, our models achieve the highest classification accuracy and precision for k=2, while for k=4, the highest recall and F1-score is attained. The k=4 model exhibits a lower standard deviation among different runs, indicating greater stability in its performance. By capturing more comprehensive structural and functional information from the sequences, the k=4 model learns robust representations that are less sensitive to variations in the training process. Therefore, we select the k=4 model as the optimal choice for further experimentation and integration within the FBGAN framework.

Regarding the ESM2 classifier, we observe that it stands out among the rest. This classifier not only achieves a remarkable 10% increase in accuracy compared to the FBGAN classifier but also has significantly less variation in its results. Having around 61.8K parameters (Table 1), which is comparable to the k=4 model but less than half of the FBGAN classifier’s parameters, underscores the effectiveness of transfer learning in capturing nuanced patterns within the data. Overall, in terms of performance ranking, the FBGAN classifier serves as the baseline, followed by the k=4 model, with the ESM2 classifier emerging as the top performer.

### 2.2. Performance of Generative Models

In this section, we assess the performance of the proposed models, namely FBGAN-kmers and FBGAN-ESM2, in comparison to the baseline FBGAN, as well as the state-of-the-art AMPGAN and HydrAMP. The goal is to provide a thorough analysis of the proposed models’ efficacy in AMP design, shedding light on the impact of the classifier and their comparative strengths and capabilities relative to existing methodologies. The evaluation process involves generating 5000 sequences for each model and assessing them based on all the metrics given in Section 4.3.2.

**Figure 3:**
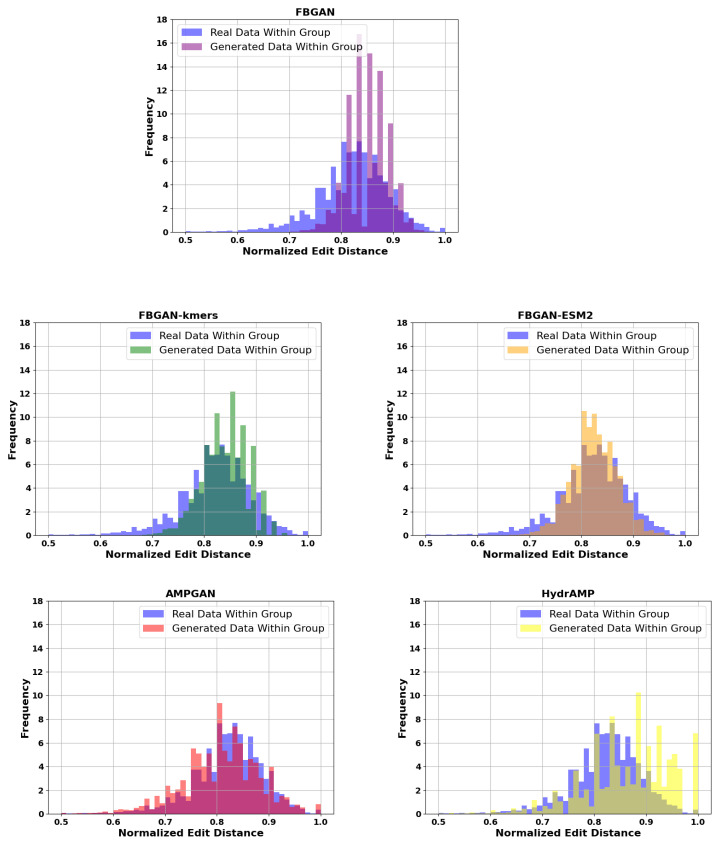
Normalized with-group edit distance distribution for real and generated data.

As illustrated in Figure 3, our models exhibit superior performance over FBGAN and HydrAMP in terms of normalized edit distance. Both FBGAN-kmers and FBGAN-ESM2 display a closer alignment in edit distance between the generated and real data. This underscores the effectiveness of our models in capturing the statistical essence of real data. Evidently, FBGAN-ESM2 produces sequences that are closer to the distribution of real data, emphasizing that the quality of the classifier directly impacts the statistical properties of the generated data. AMPGAN also performs remarkably well, indicating its competitiveness in this metric.

Table 3 presents the diversity and similarity percentages among the generated sequences. It is noteworthy that FBGAN-kmers demonstrates a similarity level comparable to FBGAN, whereas FBGAN-ESM2 exhibits a 5.9% lower similarity. This difference highlights the distinct capabilities of FBGAN-ESM2, attributed to its integration of the ESM2 classifier, which enhances its ability to generate diverse sequences with diminished similarity. In addition, AMPGAN performs on par with FBGAN-ESM2, indicating comparable similarity percentages despite differences in their underlying architectures. This implies that both models adeptly grasp the intricacies of sequence generation, albeit employing different mechanisms. Regarding diversity, Table 3 indicates that all models, except HydrAMP, produce unique sequences. However, despite its tendency to generate recurring sequences, HydrAMP surprisingly shows the lowest sequence similarity. Plausible reasons for this behavior include potential overfitting or a bias towards specific motifs within HydrAMP’s generation process. Possible explanations for this occurrence could involve the likelihood of overfitting or a preference towards particular motifs inherent in the generation process of HydrAMP.

Furthermore, the effectiveness of each generative model was verified using the $CAMP_{R4}$ server [19], an external platform equipped with diverse classifiers such as Support Vector Machine (SVM), Random Forest (RF), and Artificial Neural Network (ANN) models for AMP prediction. Table 4 outlines the classifier performance in predicting the percentage of generated AMPs among the 5000 sequences produced by the generative models. It is evident that the performance varies across different classifiers. Specifically, for the RF classifier, AMPGAN outperforms the other models by a significant margin. However, when considering the SVM and ANN classifiers, FBGAN-ESM2 emerges as the top-performing model. This suggests that evaluating the effectiveness of the generative models may be influenced by the underlying classifier used for AMP prediction. Moreover, the findings reveal that the RF classifier identifies a greater proportion of sequences as AMPs across all generative models. Considering the average performance across all classifiers, AMPGAN and FBGAN-ESM2 demonstrate comparable average prediction rates; however, AMPGAN exhibits a significantly higher standard deviation.

**Figure 4:**
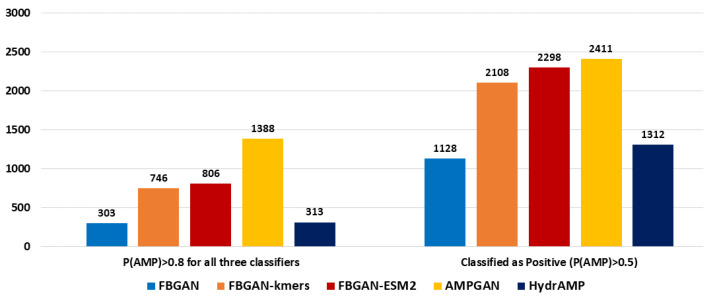
Fraction of generated peptides classified as positive by every classifier of CAMP_*R*4_ (RF, SVM, and ANN) with probabilities P(AMP)>0.5 (right) and P(AMP)>0.8 (left), respectively.

Figure 4 offers insights into the proportion of peptides classified as AMPs by RF, SVM, and ANN concurrently, with probability P(AMP)>0.5 shown in the right graph and P(AMP)>0.8 in the left one, respectively. It is important to note that the RF classifier achieves 86.5% accuracy, followed by SVM at 84.1% and ANN at 82.2% in classifying real AMPs on the CAMP_*R*4_ database [19]. Therefore, inherent biases or limitations within these classifiers may impinge upon the accuracy, therefore necessitating cautious interpretation of the results. Notably, FBGAN-kmers and FBGAN-ESM2 demonstrate superior performance over FBGAN in both total AMP generation and the production of AMPs with high probability (P(AMP)>0.8). This underscores the significance of classifier performance in enhancing both the quantity and quality of generated AMPs. On the other hand, while AMPGAN and FBGAN-ESM2 yield a comparable number of AMPs overall, AMPGAN stands out for its capacity to generate AMPs with high prediction quality (P(AMP)>0.8).

**Figure 5:**
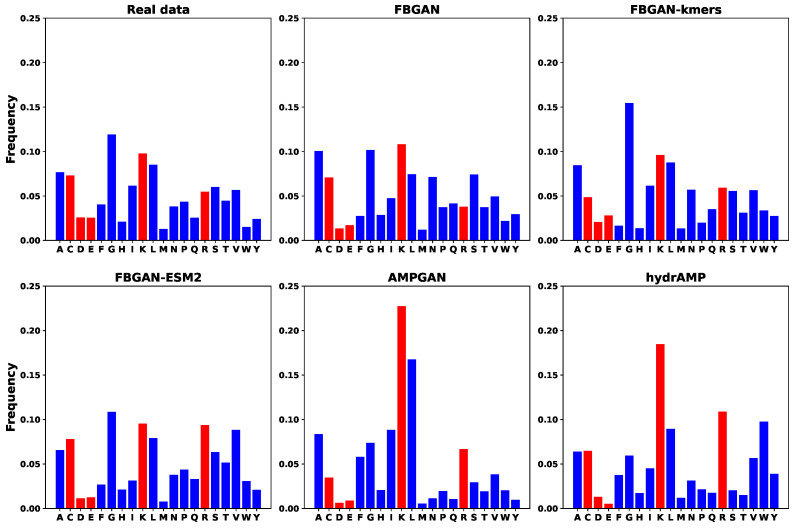
Amino acid composition for each model. The red bars mark amino acids contributing to charge, hydrophobicity, amphiphilicity, and secondary structure.

**Figure 6:**
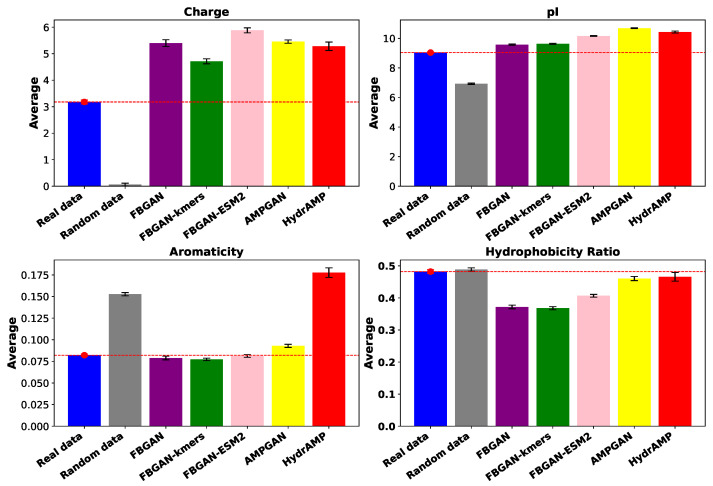
Average physiochemical features. The red dashed horizontal line indicates the average value of each feature of the real AMPs.

Finally, AMPs with P(AMP)>0.8 are selected for each generative model, and the amino acid composition and several physiochemical features like charge, pI, aromaticity, and hydrophobicity are calculated. The analysis of amino acid composition across each model is provided in Figure 5. When compared to real protein sequences, each model exhibits unique amino acid frequency distributions, suggesting differential capture of sequence features. While amino acids A, G, and L consistently emerge as prevalent across all models, notable differences exist in the frequencies of other amino acids. For example, models like AMPGAN and HydrAMP demonstrate elevated frequencies of amino acids K and R, potentially indicating a focus on sequences with higher charge or involvement in secondary structure. Conversely, amino acids C, D, and E, associated with charge, as well as K and R, contributing to hydrophobicity and amphiphilicity, exhibit relatively lower frequencies across most models. These differences may reflect the diverse training data and model architectures employed, highlighting the need for careful consideration of model-specific characteristics when interpreting predictions or generating synthetic sequences. Moreover, we utilize the Kullback–Leibler Divergence (KLD) scores to quantify the statistical closeness of amino acid compositions between real and generated protein sequences. Lower KLD scores, such as those observed for FBGAN (0.065), FBGAN-kmers (0.068), and FBGAN-ESM2 (0.061), suggest relatively small differences in amino acid composition between the real and generated sequences. This indicates that the generated sequences closely resemble the real ones in terms of their amino acid composition. On the other hand, higher KLD scores, such as those for AMPGAN (0.24) and hydrAMP (0.21), indicate that the generated sequences diverge more from the amino acid composition of the real sequences. Figure 6 depicts the average value for these features for every generative model, real AMP sequences, and randomly generated protein sequences. The higher charge of the generated sequences compared to the average for real ones suggests that the generated sequences possess an elevated proportion of basic amino acids, potentially enhancing their electrostatic interactions with bacterial membranes, a key factor in antimicrobial activity. Notably, FBGAN-ESM2 exhibits the highest charge among the generative models studied, indicating its potential for producing AMPs with strong antimicrobial properties. Considering pI, AMPGAN achieves higher average values compared to the rest, with HydrAMP and FBGAN-ESM2 following. As the pI of a molecule is the pH at which it carries no net electrical charge due to the equal numbers of positive and negative charges present on its constituent amino acids, these models might exhibit increased solubility under physiological pH conditions, which could enhance the stability and bioavailability of the peptide. HydrAMP stands out with remarkably elevated average aromaticity compared to real data, contrasting with the other models whose performance aligns closely with real data in this aspect. This notable difference is very close to that observed in random sequences, suggesting a potential lack of meaningful correlation between its generated sequences and real data in this particular aspect. Lastly, the hydrophobicity ratio is notably lower for FGBAN-based models. This divergence suggests that peptides generated by FGBAN-based models possess a reduced hydrophobic character compared to those generated by AMPGAN and HydrAMP, possibly impacting their interaction with hydrophobic regions of bacterial membranes and, hence, their antimicrobial activity.

## 3. Discussion

The primary objective of this study is to investigate the impact of advanced classifiers on the quality and efficacy of de novo AMP design. Specifically, the study aims to ascertain whether more accurate classifiers improve AMP generation within the FBGAN framework.

The introduction of two novel alternative classifiers, based on the *k*-mers technique and transfer learning with ESM2, showcases remarkable improvements in classifier performance compared to the baseline FBGAN classifier. The hierarchical approach adopted, with incremental enhancements in classifier quality, provides valuable insights into the impact of classifier performance on AMP design efficacy. Specifically, the *k*-mers-based classifier demonstrated superior performance over the baseline FBGAN classifier, achieving higher classification accuracy, precision, recall, and F1-score across various *k*-values. However, it was observed that the performance of the k=5 model was suboptimal, potentially due to overfitting resulting from excessive model complexity. This underscores the importance of selecting an optimal *k*-value to balance model complexity and generalization ability. The k=4 model was selected as a robust classifier with minimum standard deviation among independent runs. Furthermore, the ESM2 classifier emerged as the top performer, achieving a substantial increase in accuracy compared to the baseline FBGAN classifier of 10% while maintaining a significantly lower parameter count. This highlights the effectiveness of transfer learning in capturing nuanced patterns within AMP sequences.

Integration of these advanced classifiers into the FBGAN architecture leads to major performance improvements in AMP generation. Notably, both FBGAN-kmers and FBGAN-ESM2 outperformed the baseline FBGAN model and exhibited closer alignment in edit distance between generated and real data, indicating their effectiveness in capturing the statistical essence of real-world AMP sequences. Specifically, FBGAN-ESM2 produced sequences that were closer to the distribution of real data, emphasizing the impact of the classifier on the statistics of generated sequences. Furthermore, both FBGAN-kmers and FBGAN-ESM2 exhibited superior performance compared to state-of-the-art methods such as HydrAMP. They also demonstrated comparable effectiveness to AMPGAN. This comparative analysis underscores the critical importance of classifier quality in terms of accuracy, with FBGAN-kmers and FBGAN-ESM2 consistently surpassing the baseline FBGAN model, with FBGAN-ESM2 emerging as the most promising among them.

The evaluation using the CAMP_*R*4_ platform, which utilizes three AMP classifiers, RF, SVM, and ANN, reveals interesting trends in AMP prediction across different classifiers. AMPGAN emerged as the top performer, particularly with the RF classifier, showcasing its effectiveness in generating sequences with AMP characteristics. On the other hand, FBGAN-ESM2 demonstrated remarkable consistency across multiple classifiers, indicating its robustness and reliability in producing high-quality AMP candidates. This consistency underscores the effectiveness of transfer learning that enables the extraction of rich and meaningful features, enhancing the overall quality and reliability of generated AMP candidates. Moreover, FBGAN-kmers and FBGAN-ESM2 demonstrated superior performance over FBGAN in both total AMP generation and the production of high prediction quality AMPs (P(AMP)>0.8). This once again underscores the significance of classifier performance in enhancing both the quantity and quality of generated AMPs. While AMPGAN and FBGAN-ESM2 yielded a comparable number of AMPs overall, AMPGAN stood out for its proficiency in generating AMPs with high prediction quality (P(AMP)>0.8).

Further examination of the physiochemical features of high-quality generated AMPs provided valuable insights into their potential antimicrobial efficacy. Specifically, FBGAN-ESM2 demonstrated a higher charge, indicating potentially enhanced electrostatic interactions with bacterial membranes, while AMPGAN exhibited greater solubility at physiological pH, potentially improving stability and bioavailability. When considering parameters like pI and aromaticity, FBGAN-ESM2 achieved results comparable to AMPGAN that closely aligned with real data, suggesting similar solubility at physiological pH, potentially enhancing peptide stability and bioavailability. Finally, the hydrophobicity ratio was slightly lower for FBGAN-based models, particularly FBGAN and FBGAN-kmers. This implies that peptides generated by FBGAN-based models may possess reduced hydrophobicity compared to those generated by AMPGAN and HydrAMP, potentially impacting their interaction with hydrophobic regions of bacterial membranes and, thus, their antimicrobial activity.

## 4. Materials and Methods

### 4.1. Datasets

To provide a fair comparison, this work utilizes the datasets established by Gupta and Zhou [6]. The datasets consist of diverse peptide sequences sourced from various organisms. These datasets serve as valuable resources for training and evaluating the proposed classifiers within the FBGAN framework.

**AMP Classification Dataset.** The AMP classification dataset comprises 5200 protein sequences, with 2600 of these being AMPs that have been retrieved from the APD3 database [24]. The other 2600 peptides are randomly selected protein sequences from UniProt [25] with lengths ranging from 10 to 50 amino acids.

**GAN Dataset.** The dataset comprises 3655 protein sequences sourced from the UniProt database with lengths between 5 and 50 amino acids and sequence similarity greater than 0.5. This selection process aimed to capture a broad spectrum of protein lengths while ensuring computational feasibility and effective learning within the generative models.

#### Dataset Preparation and Standardization

The amino acid sequences were converted into complementary DNA sequences. This conversion involved assigning each amino acid to a corresponding codon, with considerations for redundancy and randomness when multiple codons mapped to a single amino acid. Furthermore, to standardize the dataset and ensure uniformity in sequence length, all sequences were padded to a length of 156, which represented the maximum possible sequence length within the dataset.

### 4.2. Protein Sequence Representation and Classification

In this section, we present the methodology for representing protein sequences, a crucial step in training classifiers for AMP identification. Efficiently capturing the structural and functional information encoded within protein sequences is paramount for accurate classification. We explore two distinct techniques for representing proteins, each with its unique approach to encapsulating the essential features of protein sequences. These representations aim to facilitate the classification of antimicrobial peptides with high accuracy.

#### 4.2.1. Proposed Data Representation via K-Mers Technique

In various bioinformatics applications, including genome assembly, sequence alignment, and functional annotation, the use of *k*-mers is crucial [22]. *k*-mers can provide insights into various genomic features and functions, such as identifying overlaps between sequencing reads, predicting the function of uncharacterized sequences, and inferring evolutionary relationships between different sequences. More specifically, *k*-mers are contiguous subsequences of length *k* derived from biological sequences such as DNA. The number of possible *k*-mers in a sequence is determined by the length of the sequence and the value of *k*. Specifically, for a sequence of length L, the number of *k*-mers is L−k+1. Each *k*-mer can be composed of any combination of the four DNA nucleotides A, T, G, and C. Therefore, the total number of possible *k*-mers is $4^{k}$.

The significance of *k*-mers can vary depending on the value of *k* chosen. The choice of *k* value can impact the interpretability of the model. A larger *k* value may capture more complex patterns in the data, but these patterns may be harder to interpret and may not necessarily correspond to biologically relevant features. In contrast, a smaller *k* value may capture simpler patterns that are easier to interpret but may not be sufficient to capture the full complexity of the data. In this study, we chose to evaluate models with *k* values ranging from 2 to 5 to assess the trade-off between model complexity and interpretability. In more detail, the different levels of resolution and information of the selected *k* values are namely,

- Dinucleotides or 2-mers (k=2) represent pairs of adjacent amino acids in the protein sequence. This captures short-range interactions between amino acids and provides insights into local structural motifs such as helices and turns.
- Trinucleotides or 3-mers (k=3), also known as a codon, are sequences of three nucleotides that encode a specific amino acid during protein synthesis. The frequency of each codon in a coding sequence can indicate the level of expression or translational efficiency of a gene. Tripeptides can capture more complex local structural features, including beta turns and secondary structure elements.
- Tetranucleotides or 4-mers (k=4), can be used to infer the genomic signature of an organism, which is influenced by factors such as mutation, selection, and horizontal gene transfer [26].
- Pentanucleotides or 5-mers (k=5) provide broader coverage of local sequence patterns that may capture more diverse structural motifs and functional motifs, including protein–protein interaction sites or substrate binding sites.

**Figure 7:**
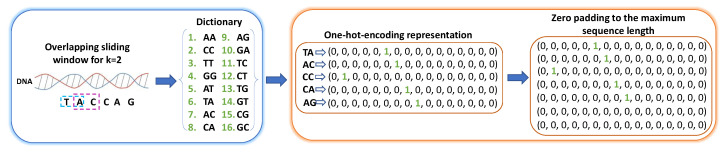
An example of the proposed DNA sequence processing and representation via the *k*-mers technique for k=2 and maximum sequence length equal to 7.

The procedure for converting DNA or protein sequences into binary vectors, a machine learning-compatible format, involves two main steps that are described below and depicted in Figure 7.

**Generating the k-mers Dictionary**. The *k*-mers are identified using an *overlapping sliding window* approach, where a window of fixed length *k* is moved along the sequence one position at a time. This process is repeated for all positions in the sequence, resulting in a collection of *k*-mers. Then, a dictionary is created containing all possible *k*-mers that can be derived from the input sequences. This dictionary comprises $4^{k}$ unique *k*-mers, each of which is assigned a unique index that is later used for one-hot-encoding.

**One-hot-encoding and zero-padding.** This is a method for representing categorical data as binary vectors. In this case, each *k*-mer in the sequence is represented as a binary vector of length equal to the number of *k*-mers ($1\times 4^{k}$) in the dictionary. The vector has a value of 1 in the position corresponding to the index of the *k*-mer in the dictionary and a value of 0 in all other positions. Finally, to ensure that all input sequences have the same length, the one-hot-encoded representation is zero-padded to reach the length of the longest sequence in the dataset, which is $L_{max}-k+1$. Specifically, zero vectors of size $1\times 4^{k}$ are appended to the input representation to ensure uniformity in length, and that is standardized and amenable for use in diverse machine learning models.

The combination of the two aforementioned methods is a novel representation of DNA sequences completely different from the representation of Gupta and Zou, who utilize a sequence of numbers based on the index of the DNA bases where {A:1, *T*:2, *C*:3, *G*:4}. For example, the sequence “TACCAG” given in Figure 7 would be simply presented as [2,1,3,3,1,4]. Additionally, one-hot encoding is not employed, and the sequences are also padded to the longest sequence length in the batch. Although this approach can be interpreted as a *k*-mers-based method with k=1, it is important to note the differences in encoding compared to the *k*-mers-based architectures proposed in this study.

#### 4.2.2. Transfer Learning with ESM2

In this study, we employed ESM2-t12, a transformer-based language model with 12 layers and 35M parameters, designed for protein sequence analysis. The main reason for selecting ESM2-t12 against other larger ESM2 variants is that it strikes a balance with a relatively modest embedding size and a manageable number of parameters, making it well-suited for our computational setup within the GAN architecture and aligns with the capacity of our available computational resources. For context, the subsequent model ESM2-t33 possesses 650 million parameters, which is significantly greater than the parameter count of ESM2-t12. This significant increase underscores the computational demands associated with other ESM2 variants and reinforces the suitability of ESM2-t12 for our study’s objectives and computational constraints.

**Figure 8:**
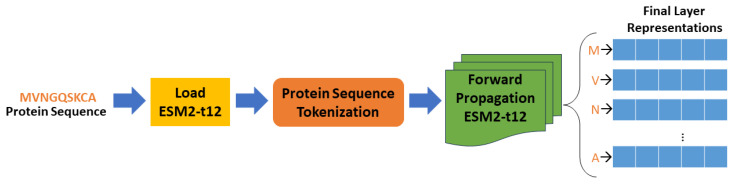
Overview of the transfer learning process using ESM2-t12 Model.

Transfer learning within the ESM2-t12 framework involves leveraging a pre-trained neural network to discern meaningful representations or embeddings from input protein sequences. Trained through unsupervised learning on a substantial corpus of protein sequences, the model adeptly captures intricate features and patterns within the sequences. Employing the self-attention mechanism and transformer architecture [27], the model effectively captures long-range dependencies and intricate relationships within the sequences, resulting in rich and informative embeddings. In our methodology, presented in Figure 8, we begin by loading the pre-trained ESM2-t12 model and tokenizing the protein sequences using the model’s tokenizer. These tokenized sequences are then fed into the pre-trained model that outputs the embeddings. These embeddings, which represent the final layer representations of the model, encapsulate both semantic and structural information inherent in the protein sequences. To obtain a fixed-size representation for each sequence, we compute the mean of the embeddings across the sequence length dimension. This process yields a vector representation, or embedding, for each protein sequence, succinctly capturing its fundamental characteristics.

#### 4.2.3. Experimental Setup

This section provides a detailed description of the neural network frameworks utilized for each representation as well as their parameter settings (Table 1). Each framework is developed using PyTorch, an open-source machine learning framework in Python. The implementation on a desktop computer equipped with NVIDIA’s GPU model Quadro P4000 demonstrates the practical feasibility of our approach.

#### 4.2.4. Network Architecture and Parameter Tuning

**FBGAN Classifier:** The classifier within the FBGAN architecture is composed of two GRU layers. The output from the second GRU layer, obtained at the final time step, is fed into a dense output layer. The number of neurons in this dense layer is determined by the number of output classes minus one. A sigmoid activation function is applied to the dense layer to compute output probabilities corresponding to the positive class. Dropout regularization is incorporated into both GRU layers to prevent overfitting. Training of the classifier is conducted using the Adam optimizer, which optimizes the binary cross-entropy loss function. Minibatch gradient descent is employed for training. Lastly, the neural network’s input is a sequence of numbers based on the index of the DNA bases where {A:1,T:2,C:3,G:4}.

**Figure 9:**
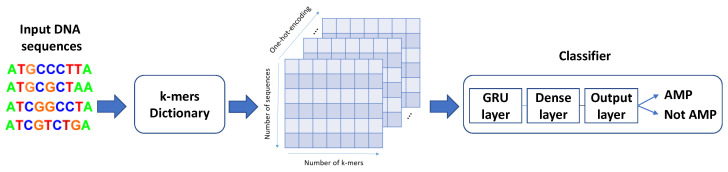
General proposed *k*-mers-based architecture with a Gated Recurrent Neural Network (GRU).

***k*-mers-based Classifier:** The architecture, provided in Figure 9, and includes a GRU and a fully connected layer. For classification, we employed a well-established approach using SoftMax activation, which provides probabilities for each output class. The optimization process utilized the Adam optimizer with a constant learning rate, along with exponential decay rates for the first and second moment estimates set to $\beta_{1}=0.9$ and $\beta_{2}=0.999$, respectively (default values). Categorical cross-entropy and categorical accuracy were employed as loss function and performance measures during the training process, respectively. Additionally, an early stopping criterion was used to terminate the training process when the validation loss did not decrease for 60 consecutive epochs. Overfitting occurs when the model performs well on the training data but poorly on the validation or test data. By monitoring the validation loss and terminating the training process when the loss does not decrease for a certain number of epochs, the approach ensures that the model does not memorize the training data but instead learns to generalize to new data. Finally, as discussed in Section 4.2.1, the neural network’s input is a one-hot-encoded representation of the *k*-mers.

**Figure 10:**
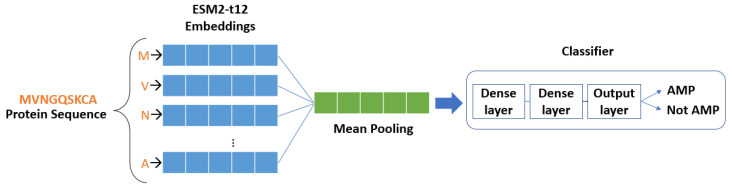
General proposed transfer learning-based architecture with Multilayer Perceptron (MLP).

**Transfer learning-based Classifier:** An MLP classifier, described in Figure 10, is designed for the classification of the protein sequence embeddings derived via the process of transfer learning from the ESM2 model. It consists of two fully connected layers, where the first layer employs ReLU activation followed by dropout regularization to prevent overfitting, and the second layer produces the final output logits. The model is trained using the Adam optimizer with a constant learning rate, along with exponential decay rates for the first and second moment estimates set to $\beta_{1}=0.9$ and $\beta_{2}=0.999$. A cross-entropy loss function is employed, and training is performed over 30 epochs. During training, the model with the lowest validation loss is saved. Finally, the saved model is evaluated on a separate test set to assess its performance in terms of accuracy. This classifier offers flexibility in adjusting parameters such as hidden layer size, dropout probability, and learning rate, making it suitable for various protein sequence classification tasks.

#### 4.2.5. Hyperparameter Tuning

For each of the proposed classifiers, we conducted extensive experimentation and fine-tuned the hyperparameters to maximize the model’s accuracy. Specifically, a grid search method was used to determine the optimal values for the number of hidden states, learning rate, batch size, and dropout. Considering the *k*-mers-based classifier, this process was repeated for each *k*. The parameter space for each hyperparameter was as follows:

- Number of layers: {1,2,3}
- Hidden states: {32,64,128,256}
- Learning rate: $\left\{10^{-5},10^{-4},10^{-3},10^{-2},10^{-1}\right\}$
- Batch size: {8,16,32,64}
- Dropout: {0.0,0.1,0.2,0.3,0.4,0.5}

The architecture and selected values for each proposed classifier, as well as the classifier incorporated in FBGAN, are depicted in Table 1. It is worth noting that these *k*-mers-based models have significantly fewer parameters than FBGAN classifier, which contains a substantial 198.9 K parameters. Specifically, as shown in Table 1, the k=2 model has 15.8 K parameters, the k=3 model has 25.0 K parameters, and the k=4 model has 61.9 K parameters. The *k*-mers-based models for k=2,3 and 4, achieve high performance with much smaller model architectures, using less than half the number of parameters compared to FBGAN classifier. The analysis of parameter counts not only demonstrates the efficiency of the *k*-mers-based models but also underscores the significance of minimizing unnecessary model complexity. This insight underscores the delicate balance between model complexity and generalization capability, underscoring the importance of selecting an optimal *k*-value to ensure that our models remain accurate, robust, and reliable.

It is noteworthy that, compared to the FBGAN classifier, the proposed transfer learning and *k*-mers-based classification schemes exhibit a notable reduction in size for k=2,3, and 4, whereas for k=5, our model is comparable in size. One potential advantage of our models is that they are more efficient in terms of memory usage and computational resources required. With fewer parameters, our models can be faster and more easily deployable than the competitive model. Additionally, our models may be less prone to overfitting as they have fewer parameters to learn from the data.

#### 4.2.6. Classification and Performance Metrics

To align with the setup of the FBGAN classifier, we divided the data in a stratified manner into three non-overlapping sets: 60% for training, 20% for validation, and 20% for testing. The model’s performance is measured based on the lowest validation loss achieved during training. After identifying the best-performing model, we evaluated it on the test set to determine the final performance of the method. We repeated the classification procedure 20 times using random data splits and reported the average performance, along with the standard deviation. To assess the performance of the proposed architecture, we measured its classification accuracy, precision, recall, and F1-score. These metrics provide a comprehensive view of the model’s ability to correctly classify AMP sequences since they take into account both true and false positive and negative predictions.

A noteworthy aspect to consider is that the performance of the FBGAN classifier is only reported for a single run, while our evaluation involves conducting multiple runs and computes the average performance. To provide a fair comparison, since the authors provide their code [6], we ran every model 20 times, using random splits, and reported the average performance. This can mitigate the impact of random fluctuations in the training and testing data, therefore obtaining a more reliable estimate of the model’s true performance.

### 4.3. Feedback GAN Architecture

The Feedback GAN (FBGAN) is an extension of the Wasserstein Generative Adversarial Network with Gradient Penalty (WGAN-GP) [28] that incorporates a feedback mechanism through a classifier to enhance sample relevance with respect to the antimicrobial property. The FBGAN architecture consists of three components: a generator *G* and a discriminator *D*, similar to traditional GANs, and an additional classifier. The generator *G* produces synthetic data samples *x* from a latent noise vector *z*, while the discriminator *D* evaluates the authenticity of these samples by assigning a probability D(x) indicating the likelihood that *x* comes from the real data distribution.

The loss function of FBGAN is formulated in terms of the Wasserstein distance [29] between the distributions of real data samples $P_{r}$ and generated samples $P_{g}$ that is defined as:

$$W\left(P_{r},P_{g}\right)=\underset{{∥D∥}_{L}\leq 1}{\sup}E_{x∼P_{r}}\left[D\left(x\right)\right]-E_{x∼P_{g}}\left[D\left(x\right)\right]$$

where ${∥D∥}_{L}$ is the Lipschitz “norm” of the discriminator *D*, and $E_{x∼P_{r}}\left[D\left(x\right)\right]$ and $E_{x∼P_{g}}\left[D\left(x\right)\right]$ are the mean output of the discriminator when given real data *x* and generated data, respectively. The efficient implementation of Wasserstein loss is performed through the incorporation of a gradient penalty term. This penalty term enforces Lipschitz continuity, therefore stabilizing training and enhancing the quality of generated samples [28]. The overall objective function can be expressed as:

$$\min_{G}\max_{D}\left(E_{x∼P_{r}}\left[D\left(x\right)\right]-E_{x∼P_{g}}\left[D\left(x\right)\right]\right)+\lambda E_{\hat{x}∼P_{\hat{x}}}\left[(∥\nabla_{\hat{x}}D\left(\hat{x}\right){{∥}_{2}-1)}^{2}\right]$$

where λ is a hyperparameter that controls the strength of the gradient penalty, $\hat{x}$ is sampled uniformly along straight lines between pairs of real and generated samples, and $P_{\hat{x}}$ is the distribution of these interpolated samples.

Finally, the classifier plays a crucial role in the FBGAN pipeline, evaluating the quality and biological relevance of the generated sequences. It is essential to clarify that the classifier operates independently of the loss function optimization process. Unlike the generator and discriminator, which directly contribute to the adversarial training dynamics, the classifier serves as an external evaluation tool. This means that the classifier’s assessments are not incorporated into the formal optimization objectives of the FBGAN model. Instead, it functions like a black box, providing feedback on the generated sequences without directly influencing the training process. The classifier can take various forms, ranging from automated algorithms to human experts capable of assessing biological properties. Regardless of its specific implementation, the classifier’s feedback guides the training process by identifying sequences that meet predefined criteria, such as AMPs, and incorporating them into the discriminator’s dataset for further training (Figure 1).

#### 4.3.1. Feedback-Loop Training

During the training procedure, the learning rate is set at 0.0001 to facilitate stable convergence, while a batch size of 64 balances computational efficiency with gradient accuracy. Sequences are constrained to a maximum length of 156 nucleotides (50 amino acids), affording flexibility in generating sequences of varying lengths. The hidden dimensionality within both the generator and discriminator is established at 512, striking a balance between model complexity and representational capacity. Throughout 150 epochs, the model undergoes iterative refinement, alternating updates between the discriminator and generator networks. For each iteration, the discriminator is subjected to 10 training steps per epoch while the generator undergoes a single update. The computation of losses is based on the Wasserstein distance between the distributions of real and generated sequences, augmented by a gradient penalty term with a coefficient of 10. Updates to the network parameters are performed via the Adam optimizer. The evaluation of generated sequences occurs every epoch throughout the training process. The final layer of the generator employs a Gumbel SoftMax operation with a temperature of 0.75, replacing the traditional SoftMax, allowing for stochastic sampling during sequence generation. When sampling from the generator, the argmax of the probability distribution is taken to output a single nucleotide at each position. After sampling 960 sequences (15 batches) from the generator, the external pre-trained classifier is employed to assess their quality and biological relevance. As shown in Figure 1, this classifier evaluates the protein sequences, focusing on identifying those with a high probability (≥0.8) of being AMPs. Such sequences are then designated to replace the positive real data used for training the discriminator.

#### 4.3.2. Evaluation of AMP Generative Models

The FBGAN outperforms vanilla WGAN in generating biologically valid peptides [6]. To ensure the fidelity of FBGAN’s performance with various classifiers, the generated sequences are further assessed. Initially, sequences are filtered based on specific criteria to ensure the correct gene structure, beginning with the canonical start codon ’ATG’ and ending with one of three canonical stop codons (’TAA’, ’TGA’, ’TAG’). These constraints are outlined in FBGAN evaluation protocols; however, in this study, before any further assessment, the ’ATG’ codon is removed from every generated sequence to prevent potential bias introduced by the artificial inclusion of the start codon. The intra-edit distance between the selected generated sequences and real genes is examined as a measure of sequence fidelity. In addition, we delve into the aspects of sequence diversity and similarity, which are pivotal in understanding the breadth and uniqueness of the generated dataset. Sequence diversity reflects the variety of sequences produced by the generator, indicative of its ability to comprehensively explore the sequence space. On the other hand, sequence similarity serves as a critical metric for evaluating the fidelity of the generated sequences to real-world data. This involves quantifying the resemblance between synthetic sequences and authentic biological sequences. One of the algorithms commonly used to assess sequence similarity is the Needleman-Wunsch algorithm [30]. This algorithm performs global sequence alignment, finding the optimal alignment between two sequences based on a scoring scheme that considers matches, mismatches, and gaps. By computing similarity scores, such as sequence alignment scores or sequence identity percentages, the extent to which the generated sequences mimic known biological sequences can be assessed.

Moreover, the proposed models FBGAN-kmers and FBGAN-ESM2, as well as the original FBGAN, are evaluated against the leading generative frameworks for AMP de novo design: AMPGAN [7] and HydrAMP [9]. AMPGAN represents a state-of-the-art bidirectional conditional GAN [31,32] framework specifically tailored for AMP de novo design. Its architecture utilizes generative adversarial networks (GANs) to sample AMP sequences with unprecedented diversity. On the other hand, HydrAMP is a novel conditional variational autoencoder (cVAE) [33] model designed for AMP generation. Unlike traditional approaches, HydrAMP integrates advanced machine learning techniques with domain-specific knowledge to capture the complex relationship between peptide structure and function.

The evaluation process includes generating 5000 sequences for each proposed model and FBGAN. To evaluate AMPGAN and HydrAMP, we utilize the publicly available generated sequences provided by the authors of HydrAMP along with their code. Considering HydrAMP, we select 5000 sequences produced from the unconstrained simulation where the model is trained to generate peptides de novo. Moreover, in contrast to HydrAMP, we do not perform biological filtering on the generated peptides. In particular, for each model, we randomly select 5000 generated peptides of length less than 50 amino acids and assess them based on the metrics discussed previously in this section. This analysis allows for a thorough comparison of the proposed models with FBGAN, AMPGAN, and HydrAMP, providing insights into their performance in antimicrobial peptide design. Furthermore, the quality of every generative model is further validated using the $CAMP_{R4}$ server [19], an independent platform equipped with diverse classifiers such as Support Vector Machine (SVM), Random Forest (RF), and Artificial Neural Network (ANN) models for AMP prediction. By subjecting the FBGAN-generated sequences to the scrutiny of these classifiers, we aim to obtain additional assurance regarding their authenticity as AMPs. This orthogonal validation procedure not only corroborated the findings from our primary assessments but also bolstered confidence in the fidelity of the FBGAN-generated sequences as biologically relevant peptides. Such validation efforts are crucial for ensuring the reliability and utility of computational methods in peptide design and discovery.

Finally, AMPs exhibit diverse physiochemical properties that contribute to their antimicrobial activity [34,35]. Therefore, the generated peptides classified simultaneously as AMPs by RF, SVM, and ANN with high probability (P(AMP)>0.8) are further scrutinized for their similarity to real AMP sequences, leveraging physiochemical features such as charge, isoelectric point (pI), aromaticity and hydrophobicity. AMPs often possess a net positive charge due to an abundance of basic amino acids such as arginine and lysine, which facilitates their interaction with negatively charged bacterial membranes [36]. The isoelectric point (pI) of AMPs, representing the pH at which they carry no net charge, influences their solubility and stability. Aromaticity, referring to the presence of aromatic amino acids like phenylalanine and tryptophan, contributes to the peptide’s structural stability and interaction with lipid membranes. Hydrophobicity, characterized by the presence of hydrophobic residues such as leucine and alanine, affects the peptide’s membrane permeabilization and overall antimicrobial potency. Collectively, these physiochemical features play crucial roles in determining the efficacy and selectivity of AMPs against microbial pathogens.

## 5. Conclusions

The findings of this study underscore the effectiveness of advanced classifiers within generative models for AMP design. The integration of more accurate classifiers not only improves the accuracy and realism of generated sequences but also enhances the potential antimicrobial efficacy of the designed peptides. The proposed generative models, FBGAN-kmers and FBGAN-ESM2, outperform HydrAMP overall. Additionally, FBGAN-ESM2 demonstrates comparable performance to the state-of-the-art AMPGAN. Future research will prioritize further optimization of the proposed classifier-driven generative models, incorporating the classifiers’ impact on the loss function during training.

## Figures and Tables

**Table 1:** Network parameters for the proposed *k*-mers-based, transfer learning-based, and FBGAN classifier.

	FBGAN	*k*-Mers-Based Classifier				ESM2-Based
	Classifier	*k* = 2	*k* = 3	*k* = 4	*k* = 5	Classifier
**Input size**	4	16	64	256	1024	480
**Layers**	2 GRUs and 1 Dense	1 GRU and 1 Dense	1 GRU and 1 Dense	1 GRU and 1 Dense	1 GRU and 1 Dense	2 Dense
**Hidden states**	128	64	64	64	64	128
**Learning rate**	0.001	0.001	0.001	0.001	0.001	0.001
**Dropout**	0.3 in both layers	0.3	0.2	0.3	0.3	0.2
**Batch size**	64	64	8	64	32	16
**Total number of parameters**	198.9 K	15.8 K	25.0 K	61.9 K	209.4 K	61.8 K

**Table 2:** Performance evaluation of the work of FBGAN classifier and the proposed classification schemes, in terms of average classification accuracy, precision, recall, and F1-score. The standard deviation among different runs is included in parentheses.

	FBGAN	*k*-Mers				ESM2
		*k* = 2	*k* = 3	*k* = 4	*k* = 5	
**Accuracy**	81.4 (7.0)	85.7 (1.4)	85.6 (1.5)	85.3 (1.0)	82.3 (7.2)	**91.5 (0.7)**
**Precision**	82.3 (2.8)	84.3 (2.1)	83.8 (1.8)	82.9 (1.9)	81.7 (5.1)	**90.6 (2.3)**
**Recall**	80.5 (18.7)	87.7 (3.1)	88.3 (3.2)	89.1 (1.8)	85.0 (18.9)	**92.7 (1.7)**
**F1-score**	79.2 (16.8)	85.8 (1.5)	85.9 (1.6)	85.9 (0.8)	80.8 (17.1)	**91.6 (0.6)**

**Table 3:** Percentage of diversity and sequence similarity.

	Diversity	Sequence Similarity
**Real Data**	98.5%	24.3%
**FBGAN**	100%	33.5%
**FBGAN-kmers**	100%	33.4%
**FBGAN-ESM2**	100%	27.6%
**AMPGAN**	100%	26.8%
**HydrAMP**	98.9%	21.7%

**Table 4:** Percentage of predicted AMPs with CAMP_*R*4_ platform equipped with Support Vector Machine (SVM), Random Forest (RF), and Artificial Neural Network (ANN) models for AMP prediction. The best performance per classifier is highlighted in bold, and the standard deviation is reported in parentheses.

	CAMP_*R*4_			
	**RF**	**SVM**	**ANN**	**Average among Classifiers**
**FBGAN**	33.5%	31.4%	32.9%	32.6% (0.8)
**FBGAN-kmers**	61.8%	53.8%	49.7%	55.1% (5.0)
**FBGAN-ESM2**	59.1%	**55.5%**	**58.9%**	57.8% (1.6)
**AMPGAN**	**64.6%**	53.8%	56.6%	**58.3% (4.5)**
**HydrAMP**	58.8%	52.6%	32.9%	48.1% (11.0)

## Data Availability

Data and the software of this work are provided on the following link: https://github.com/aretiz/de_novo_design_GAN.git.

## References

[1] Zasloff M. (2002). Antimicrobial peptides of multicellular organisms. Nature.

[2] Hancock R.E., Sahl H.G. (2006). Antimicrobial and host-defense peptides as new anti-infective therapeutic strategies. Nat. Biotechnol..

[3] Lei J., Sun L., Huang S., Zhu C., Li P., He J., Mackey V., Coy D.H., He Q. (2019). The antimicrobial peptides and their potential clinical applications. Am. J. Transl. Res..

[4] Wu Q., Ke H., Li D., Wang Q., Fang J., Zhou J. (2019). Recent progress in machine learning-based prediction of peptide activity for drug discovery. Curr. Top. Med. Chem..

[5] Zervou M.A., Doutsi E., Tsakalides P. (2023). Unleashing the Power of Artificial Intelligence for Personalised Drug Design. ERCIM News.

[6] Gupta A., Zou J. (2019). Feedback GAN for DNA optimizes protein functions. Nat. Mach. Intell..

[7] Van Oort C.M., Ferrell J.B., Remington J.M., Wshah S., Li J. (2021). AMPGAN v2: Machine learning-guided design of antimicrobial peptides. J. Chem. Inf. Model..

[8] Dean S.N., Alvarez J.A.E., Zabetakis D., Walper S.A., Malanoski A.P. (2021). PepVAE: Variational autoencoder framework for antimicrobial peptide generation and activity prediction. Front. Microbiol..

[9] Szymczak P., Możejko M., Grzegorzek T., Jurczak R., Bauer M., Neubauer D., Sikora K., Michalski M., Sroka J., Setny P. (2023). Discovering highly potent antimicrobial peptides with deep generative model HydrAMP. Nat. Commun..

[10] Goodfellow I., Pouget-Abadie J., Mirza M., Xu B., Warde-Farley D., Ozair S., Courville A., Bengio Y. (2020). Generative adversarial networks. Commun. ACM.

[11] Lee H.T., Lee C.C., Yang J.R., Lai J.Z., Chang K.Y. (2015). A large-scale structural classification of antimicrobial peptides. BioMed Res. Int..

[12] Bhadra P., Yan J., Li J., Fong S., Siu S.W. (2018). AmPEP: Sequence-based prediction of antimicrobial peptides using distribution patterns of amino acid properties and random forest. Sci. Rep..

[13] Veltri D., Kamath U., Shehu A. (2018). Deep learning improves antimicrobial peptide recognition. Bioinformatics.

[14] Yan J., Bhadra P., Li A., Sethiya P., Qin L., Tai H.K., Wong K.H., Siu S.W. (2020). Deep-AmPEP30: Improve short antimicrobial peptides prediction with deep learning. Mol. Ther.-Nucleic Acids.

[15] Burdukiewicz M., Sidorczuk K., Rafacz D., Pietluch F., Chilimoniuk J., Rödiger S., Gagat P. (2020). Proteomic screening for prediction and design of antimicrobial peptides with AmpGram. Int. J. Mol. Sci..

[16] Chung C.R., Kuo T.R., Wu L.C., Lee T.Y., Horng J.T. (2020). Characterization and identification of antimicrobial peptides with different functional activities. Briefings Bioinform..

[17] Fingerhut L.C., Miller D.J., Strugnell J.M., Daly N.L., Cooke I.R. (2020). ampir: An R package for fast genome-wide prediction of antimicrobial peptides. Bioinformatics.

[18] Lawrence T.J., Carper D.L., Spangler M.K., Carrell A.A., Rush T.A., Minter S.J., Weston D.J., Labbé J.L. (2021). amPEPpy 1.0: A portable and accurate antimicrobial peptide prediction tool. Bioinformatics.

[19] Gawde U., Chakraborty S., Waghu F.H., Barai R.S., Khanderkar A., Indraguru R., Shirsat T., Idicula-Thomas S. (2023). CAMPR4: A database of natural and synthetic antimicrobial peptides. Nucleic Acids Res..

[20] Yan K., Lv H., Guo Y., Peng W., Liu B. (2023). sAMPpred-GAT: Prediction of antimicrobial peptide by graph attention network and predicted peptide structure. Bioinformatics.

[21] Chung J., Gulcehre C., Cho K., Bengio Y. (2014). Empirical evaluation of gated recurrent neural networks on sequence modeling. arXiv.

[22] Karlin S., Altschul S.F. (1990). Methods for assessing the statistical significance of molecular sequence features by using general scoring schemes. Proc. Natl. Acad. Sci. USA.

[23] Lin Z., Akin H., Rao R., Hie B., Zhu Z., Lu W., Smetanin N., Verkuil R., Kabeli O., Shmueli Y. (2023). Evolutionary-scale prediction of atomic-level protein structure with a language model. Science.

[24] Wang G., Li X., Wang Z. (2016). APD3: The antimicrobial peptide database as a tool for research and education. Nucleic Acids Res..

[25] Consortium U. (2019). UniProt: A worldwide hub of protein knowledge. Nucleic Acids Res..

[26] Abe T., Kanaya S., Kinouchi M., Ichiba Y., Kozuki T., Ikemura T. (2003). Informatics for unveiling hidden genome signatures. Genome Res..

[27] Vaswani A., Shazeer N., Parmar N., Uszkoreit J., Jones L., Gomez A.N., Kaiser Ł., Polosukhin I. (2017). Attention is all you need. Adv. Neural Inf. Process. Syst..

[28] Gulrajani I., Ahmed F., Arjovsky M., Dumoulin V., Courville A.C. (2017). Improved training of wasserstein gans. Adv. Neural Inf. Process. Syst..

[29] Arjovsky M., Chintala S., Bottou L. Wasserstein generative adversarial networks. Proceedings of the International Conference on Machine Learning, PMLR.

[30] Likic V. (2008). The Needleman-Wunsch Algorithm for Sequence Alignment.

[31] Dumoulin V., Belghazi I., Poole B., Mastropietro O., Lamb A., Arjovsky M., Courville A. (2016). Adversarially learned inference. arXiv.

[32] Donahue J., Krähenbühl P., Darrell T. (2016). Adversarial feature learning. arXiv.

[33] Kingma D.P., Mohamed S., Jimenez Rezende D., Welling M. (2014). Semi-supervised learning with deep generative models. Adv. Neural Inf. Process. Syst..

[34] Melo M.N., Ferre R., Feliu L., Bardaji E., Planas M., Castanho M.A. (2011). Prediction of antibacterial activity from physicochemical properties of antimicrobial peptides. PLoS ONE.

[35] Kang S.J., Kim D.H., Mishig-Ochir T., Lee B.J. (2012). Antimicrobial peptides: Their physicochemical properties and therapeutic application. Arch. Pharmacal Res..

[36] Sitaram N., Nagaraj R. (1999). Interaction of antimicrobial peptides with biological and model membranes: Structural and charge requirements for activity. Biochim. Biophys. Acta Biomembr..

